# Compulsory Community Treatment Orders and health outcomes for Ma-ori in New Zealand

**DOI:** 10.1177/00048674241280918

**Published:** 2024-09-26

**Authors:** Ben Beaglehole, Chris Frampton, Giles Newton-Howes, Arahia Kirikiri, Cameron Lacey

**Affiliations:** 1 Department of Psychological Medicine, University of Otago, Christchurch, Christchurch, New Zealand; 2 Department of Psychological Medicine, University of Otago, Wellington, Wellington, New Zealand; 3 Mental Health Services, Te Whatu Ora Health New Zealand, Te Matau a Māui Hawke’s Bay, New Zealand

**Keywords:** Compulsory treatment orders, Māori, community treatment orders, coercion

## Abstract

**Background:**

We have previously analysed outcomes for all community treatment orders commenced during a 10-year period in New Zealand. Given Te Tiriti O Waitangi obligations to scrutinise health and consider equity for Māori, we completed this analysis to consider community treatment-order outcomes according to ethnicity.

**Methods:**

Ministry of Health databases provided demographic, service use and medication dispensing data for community treatment-order recipients between 2009 and 2018. As non-Māori on community treatment orders are older, less deprived and less likely to be diagnosed with a Psychotic Disorder, data were categorised according to age (<35/⩾35 years), level of deprivation (New Zealand Dep levels ⩽3, 4–6 and ⩾7) and diagnosis (Psychotic Disorder/non-Psychotic Disorder). The incidences of key outcome measures (admissions, community care, medication dispensing) were calculated for periods on/off community treatment orders for Māori and non-Māori to consider the differential impact of community treatment orders according to ethnicity.

**Results:**

Māori have high rates of community treatment order utilisation and are younger, more likely to be diagnosed with a Psychotic Disorder and spend longer receiving compulsory treatment than non-Māori. Non-Māori are more likely to receive more additional depot antipsychotic medication on–community treatment orders compared with periods off-community treatment order than Māori but other clear patterns of response distinguishing between Māori and non-Māori were not present.

**Conclusion:**

The differences between Māori and non-Māori for community treatment-order utilisation suggest the presence of structural inequity in underlying mental illness distribution and treatment provision. Māori cultural expertise at all levels of healthcare including healthcare planning and delivery is required to make advances and reduce disparity.

## Introduction

Māori are the indigenous people of Aotearoa New Zealand. The foundational document of Aotearoa is Te Tiriti O Waitangi (Treaty of Waitangi). This treaty is an agreement between Māori representatives and the British Crown outlining governance principles for New Zealand. Te Tiriti O Waitangi principles are active today. Among the principles endorsed by the New Zealand Ministry of Health (MOH; Manatū Hauora) are Tino Rangatiratanga (Māori self-determination in the design, delivery and monitoring of health and disability services), equity of health outcomes for Māori and active protection (including ensuring that the Crown and Māori are fully informed about the extent and nature of health outcomes for Māori and make efforts to reach equity) (Minister of Health, 2023).

Early studies of major mental illness in New Zealand report that rates of major mental illness were lower for Māori than non-Māori (Beaglehole, 1939). Over time, patterns have shifted and Māori now have higher rates of common mental disorders compared to non-Māori including greater severity of illness (Baxter et al., 2006). These differences persist after adjustments for socio-demographic factors (Baxter et al., 2006). Suicide rates were also lower for Māori but increased in the 1980s and are now persistently higher for Māori than non-Māori (Ora, 2024; Skegg et al., 1995). Providing some context, inequities exist between Māori and non-Māori in accessing health care. These include large shortfalls in the dispensing of medication (including psychiatric medication) for Māori (Metcalfe et al., 2018) and shorter psychiatric admissions with Māori patients being less likely to be referred for psychotherapy after discharge (Kumar et al., 2008). Interpersonal and systematic racism are prevalent for Māori accessing treatment for early psychosis and impact negatively on treatment and outcomes (Manuel et al., 2023a, 2023b).

Compulsory treatment can be provided in inpatient and outpatient settings. Compulsory community treatment orders (CTOs) enable psychiatric treatment to be provided in the community without the requirement for consent. In New Zealand, compulsory treatment is provided to patients whose mental illnesses place them at risk of serious harm to themselves or others, or are seriously diminished in their capacity to care for themselves (Pāremata). We have previously reported that 34% of CTO recipients were Māori in a 10-year study (Beaglehole et al., 2021). This is significantly higher than the percentage of Māori in the general population at the time of our study (17%) (StatsNZ, 2021) but is commensurate with the findings of higher rates of mental disorder among Māori. Other research has assessed Māori patients’ views on being the recipient of CTOs (Gibbs et al., 2004; Newton-Howes et al., 2014). Gibbs et al. reported positive views of CTOs included greater safety, security and access to care whereas negative aspects were a sense of external control and loss of choice. Newton-Howes et al. reported that there were similar views between Māori and non-Māori on CTOs including for those treated within Kaupapa Māori (Māori designed and led) services (Newton-Howes et al., 2014).

Our previous CTO research highlighted differential outcomes according to diagnosis. Patients with Psychotic Disorders had reduced frequency of admissions and reduced admission days during CTOs compared to voluntary periods whereas the opposite occurred for patients with other disorders (Beaglehole et al., 2021, 2022). We also reported that mortality was higher during CTOs despite higher rates of contact with community services and more dispensing of psychiatric medication (Beaglehole et al., 2023). Furthermore, there was marked interregional variation in CTO utilisation across New Zealand after accounting for demographic factors suggesting culture and practice differences between regions (Lees et al., 2023). In the context of the mental health statistics for Māori outlined above and Te Tiriti O Waitangi principles mandating monitoring of health and disability services and health equity, we also wished to examine CTO data for Māori. Our goal in this paper is to report and critically analyse CTO findings for Māori and consider the issue of equity.

## Methods

*Positioning statement*: BB, CF and GN are New Zealand European (Pākeha) researchers. AK (Te Whānau-ā-Apanui) and CL (Te Atiawa) are Māori researchers.

Data contained within this report was accessed from our database research reporting clinical outcomes associated with CTOs (Beaglehole et al., 2021, 2022, 2023). Three national databases provide data on all New Zealanders placed on CTOs over a 10-year period between 1 January 2009 and 31 December 2018. The data were received in de-identified form using unique identifiers. As a consequence, informed consent was not required, but ethics approval was granted by the Human Research Ethics Committee of the University of Otago (reference no. HD19/076).

The Programme for the Integration of Mental Health Data (PRIMHD) is the national mental health information collection service for the MOH, New Zealand. The PRIMHD data set records service activity and outcomes for all health consumers who receive treatment from public sector secondary care and non-governmental organisation mental health and addiction services (Ministry of Health New Zealand, 2016). PRIMHD holds data on CTO initiation and duration. PRIMHD also contains diagnostic data including the principal diagnosis for those attending secondary-care services. The New Zealand Mental Health Act excludes substance use as the primary reason for providing compulsory psychiatric treatment. Consequently, compulsory treatment of substance disorders is not addressed in this study (although a mental disorder caused by substance use such as a substance-induced psychotic disorder would still be captured in these data). Data from PRIMHD included the following:

- Date and duration of first CTO (Section 29 Mental Health Act 1992) and subsequent CTOs. Inpatients under Section 30 Mental Health Act 1992 on leave from hospital were not designated as CTOs for this analysis.
- Ethnicity (European/other, Māori, Pacifica, Asian). For the purposes of this analysis, patients were classified as Māori or non-Māori to focus attention on this distinction.
- Other demographic information including age at CTO commencement, date of birth, gender, current socio-economic status (measured in deciles through a deprivation index (Ministry of Health New Zealand, 2013) obtained using census data from area of residence with higher deciles indicating greater deprivation) and District Health Board (DHB) where domiciled.
- *DSM* IV Principal diagnostic codes to analyse findings according to diagnosis. Diagnoses were categorised according to two groups; Psychotic Disorders and non-Psychotic disorders because our previous research reported distinct outcome differences for these two groups (Beaglehole et al., 2022).
- Service use information including admissions to psychiatric institutions, duration of admissions and outpatient contacts. Phone contacts, Did Not Attend appointments and care coordination contacts such as phone calls were not included as contact appointments.

The Pharmaceutical Collection is a data warehouse containing the vast majority of dispensing data in New Zealand (Ministry of Health New Zealand, 2019). Data on psychiatric medication dispensing was requested for individuals identified in the PRIMHD sample. Data were requested using the same unique identifier to allow linkage of the data sets. Medications were categorised using the online Pharmaceutical Schedule – November 2019 (a New Zealand database of medications subsidised by the government) classes of Antidepressants (includes lithium carbonate), Antipsychotics General (comprising oral antipsychotics), Antipsychotics Depot Injections (comprising Long-acting injectable antipsychotic formulations) and Anxiolytics.

The Mortality Collection (Ministry of Health New Zealand, 2022) classifies the underlying cause of death for all deaths registered in New Zealand. For this study, we requested data for individuals identified in the PRIMHD data set and recorded in the Mortality Collection between 1 January 2009 and 31 December 2018. Mortality data was categorised according to total deaths and sub-groups according to cause of death (suicide, accidents and assaults and other medical causes). See our previous research for the International Classification of Diseases (ICD) codes detailing the different causes of death (Beaglehole et al., 2023).

This paper reports exploratory analyses of previously reported research. Our analysis compares key outcomes for Māori and non-Māori. The outcomes include the following:

- The number of psychiatric inpatient admissions/annum during CTOs compared to non-CTO periods. Admissions were required to be of greater than 48 hours to exclude brief admissions to administer depot antipsychotic medication due to refusal to accept treatment in the community.
- Number of contacts with community psychiatric services during CTOs compared to non-CTO periods.
- Rates of psychiatric medication dispensing during CTOs compared to non-CTO periods.
- Mortality rates during CTOs compared to non-CTO periods.

### Analysis

The clinical and demographic features of the study population according to ethnicity were summarised using standard descriptive statistics. The incidences of the key outcome measures were calculated for the periods on and off CTOs for Māori and non-Māori. Patients do not act as their own controls; instead, the data were aggregated according to CTO status. The grouped period for patients on CTOs was compared to the grouped period for patients off CTOs. This involved calculating the person-years represented for each individual on and off CTOs within the total study period. The total number of the relevant outcome events both on and off CTOs for each individual were then summed to calculate the rates on and off CTOs. A rate ratio (RR) was calculated using these incidences by dividing the incidence of the outcome measure on CTOs with the corresponding figure off CTOs. RRs of less than 1 report that the outcome is less likely on CTOs, and RRs greater than 1 report the outcome measure is more likely on CTOs.

As non-Māori on CTOs are older, less deprived and less likely to be diagnosed with a psychotic disorder, the data were categorised and reported according to a median split for age (<35/⩾35 years), level of deprivation (New Zealand Dep levels ⩽ 3, 4–6 and ⩾7) and diagnosis (psychotic disorder/non-psychotic disorder). The 95% confidence intervals for the incidence and ratio estimates were calculated using the standard Poisson approximation (Sahai and Khurshid, 1996). The comparisons between Māori and non-Māori are reported in text and graphical form. Where possible, comparisons are reported using non-Māori as the point of comparison to highlight the privilege that exists for non-Māori as opposed to situating disadvantage with Māori (Manuel et al., 2023a). The graphs were visually scanned for patterning across age, deprivation and ethnicity splits to detect consistent trends. SPSS version 28 was used for analysis.

Following extraction of data and statistical analysis, findings were considered using Te Tiriti O Waitangi principles to consider whether outcomes are equitable for Māori. The findings from these considerations formed the basis for the discussion.

## Results

The socio-demographic characteristics of the population placed on CTOs in New Zealand over the 10-year period between 1 January 2009 and 31 December 2018 are reported in Table 1. Thirty-four percent of this population were Māori. Non-Māori patients were older than Māori patients and were more likely to be female. Non-Māori patients were also less deprived than Māori patients. Māori patients spent longer on compulsory treatment/annum and were more likely to have a Psychotic Disorder than non-Māori patients. Correspondingly, Māori patients were less likely to be diagnosed with dementias, mood disorders and personality disorders. Twelve percent of non-Māori CTO recipients were aged less than 18 years whereas 22.1% of Māori recipients were less than 18 years.

**Table 1.** Sociodemographic characteristics of the study population.

Characteristic	Māori (*n* = 5053)	Non-Māori (*n* = 9673)
Mean age (SD)	30.2 (12.7)	37.8 (16.5)
% Male	63.4	57.8
% placed on CTO < 18 years	22.1	12.0
Mean NZ Dep (SD)	7.9 (2.3)	6.6 (2.7)
Median days spent on compulsory treatment/year (IQR)	52.4 (18.0–152.6)	36.2 (16.4–128.3)
% Dementia	1.7%	2.7%
% Psychotic disorder	63.3%	53.1%
% Bipolar 1 disorder	8.0%	10.2%
% Other bipolar disorder	1.0%	1.7%
% Depressive disorder	1.4%	3.6%
% Personality disorder	0.5%	0.8%
% Other diagnosis	4.6%	5.4%
% No diagnosis	19.5%	22.4%

**Figure 1.**
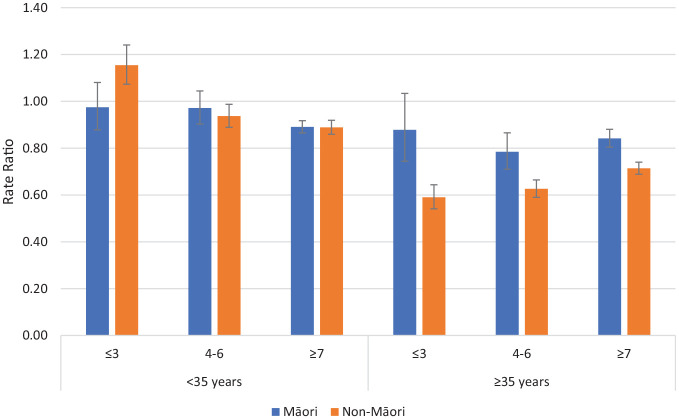
Rate ratios of admissions >48 hours on CTOs: off CTOs according to ethnicity, deprivation and age for Psychotic Disorders (error bars show 95% confidence intervals).

The RRs (for events on CTO/Off CTO) in the Psychotic Disorders group for admissions >48 hours comparing Māori and non-Māori within age and deprivation categories are reported in Figure 1. Non-Māori were more likely to be admitted on CTOs than off-CTOs in the <35 years group with low deprivation. For all other groupings, patients were less likely to be admitted on CTOs than off CTOs (RR < 1.0). The magnitude of reduced admissions on CTOs was greater for non-Māori than Māori in the ⩾35 years age bracket.

**Figure 2.**
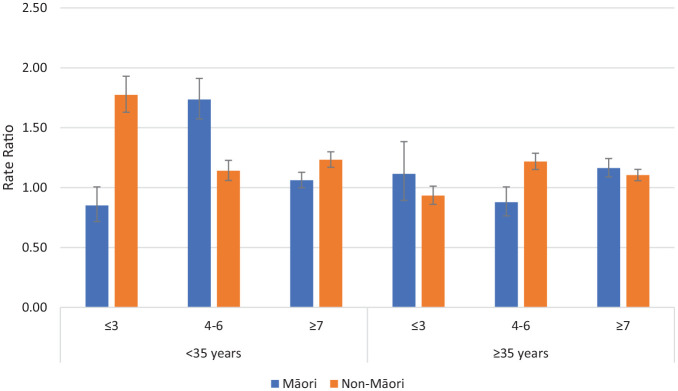
Rate ratios of admissions >48 hours on CTOs: off CTOs according to ethnicity, deprivation and age for non-psychotic disorders (error bars show 95% confidence intervals).

The RRs (for on CTO/off CTO) in the non-psychotic disorders group for admissions >48 hours comparing Māori and non-Māori within deprivation and age groups are reported in Figure 2. The majority of these deprivation/age groupings were more likely to be admitted on CTOs than off CTOs (RR > 1.0). Systematic differences between Māori and non-Māori across age and deprivation were not apparent.

The RRs for community contacts for psychotic disorders and non-psychotic disorders according to the ethnicity, deprivation and age sub-groupings are reported in Supplementary Figures 1 and 2. All RRs were greater than one meaning community contacts are more likely on CTOs than off CTOs. This was most evident for younger Māori with psychotic disorders and high deprivation (RR 4.22) but systematic differences in RR between Māori and non-Māori were not apparent.

**Figure 3.**
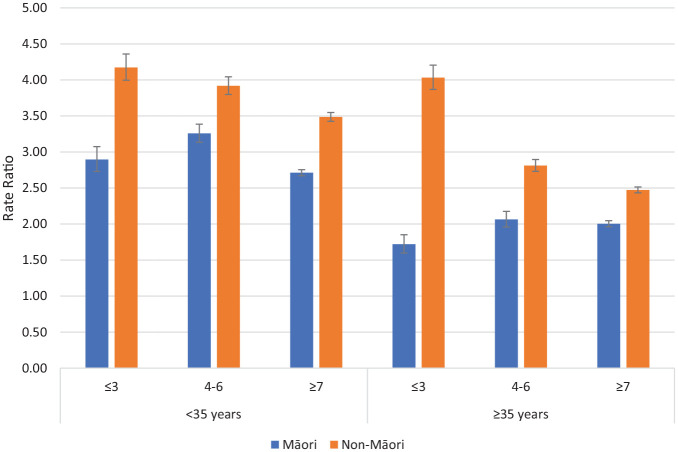
Rate ratios of depot antipsychotic dispensing on CTOs: off CTOs according to ethnicity, deprivation and age for psychotic disorders (error bars show 95% confidence intervals).

**Figure 4.**
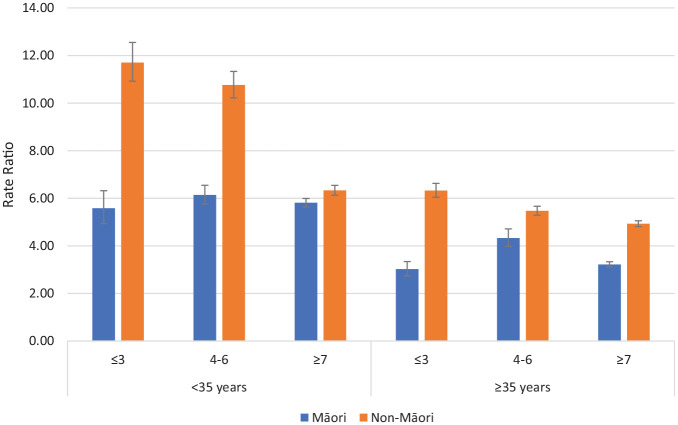
Rate ratios of depot antipsychotic dispensing on CTOs: off CTOs according to ethnicity, deprivation and age for non-psychotic disorders (error bars show 95% confidence intervals).

The dispensing ratios (RR for on CTO/Off CTO) of depot antipsychotic medication for psychotic disorders and non-psychotic disorders comparing Māori and non-Māori within deprivation, and age groupings are reported in Figures 3 and 4. For patients with psychotic and non-psychotic disorders, the RR of dispensing (on CTO/Off CTO) were greater for non-Māori than Māori for all age and deprivation groupings meaning non-Māori are more likely to receive more additional depot antipsychotic medication on CTOs compared with off CTO than Māori. Supplementary figures are provided for antidepressants, anxiolytics, depot antipsychotics, and oral antipsychotics. Generally, higher rates of dispensing occur on CTOs except for dispensing of anxiolytics to patients with non-psychotic disorders.

Three hundred and seventy-six Māori and 949 non-Māori died during the 10-year study period. This was 7.4% of the Māori cohort and 9.8% of the non-Māori cohort. Both Māori and non-Māori were more likely to die while CTOs were enacted than off-CTOs (RR: 1.71, confidence interval [CI]: 1.34–2.11 for Māori) (RR: 1.16, CI: 0.99–1.34 for non-Māori). Sample sizes were insufficient to further explore mortality by ethnicity, diagnosis, deprivation and age.

## Discussion

This study confirms that Māori receive high rates of CTOs compared to their prevalence in the general population (Beaglehole et al., 2021). We also report Māori receiving CTOs are younger, more likely to be diagnosed with a psychotic disorder and spend longer receiving compulsory treatment than non-Māori. Non-Māori are more likely to receive more additional depot antipsychotic medication on-CTOs compared with periods off-CTO than Māori. These disparities between Māori and non-Māori violate the Te Tiriti O Waitangi principle of equity of health outcomes between the two groups. The inequities also support ongoing monitoring of CTO outcomes for Māori and efforts to reduce inequity.

The high rates of CTO usage for Māori that we report appear commensurate with the higher rates of mental disorder that are now present for Māori and suggest inequity in the rate and severity of mental disorder distribution in New Zealand. In the general population, Māori are more likely to be diagnosed with schizophrenia and non-Māori are more likely to be diagnosed with bipolar disorder (Carr et al., 2023). These diagnostic differences between Māori and non-Māori were also present in our study and may reflect the underlying differences that are present in the diagnostic distribution of mental disorder in New Zealand. It has previously been posited that these diagnostic differences may relate to differential socio-environmental exposures (such as loss of land, culture or self-determination or socio-demographic disadvantage including substance use) or assessor biases (both conscious and unconscious) (Carr et al., 2023). Our data set was unable to shed further light on the underpinnings for the diagnostic variation.

Māori receiving CTOs are younger than non-Māori. The percentage of Māori who were under the age of 18 years at the time of CTO first utilisation was 22.1% compared to 12.4% non-Māori. This is a confronting figure given vulnerability is likely to be amplified in the younger aged group. Does it reflect greater severity and earlier onset of illness for Māori? Or do comorbidities such as substance use disorders that are more frequent in Māori with psychotic disorders impact negatively on illness presentations? (Carr et al., 2023). Or are other explanations such as systematic bias in access to early treatment, treatment-approach or lack of family/whānau support relevant? In addition, non-Māori placed on CTOs spent approximately 16 less days on CTOs/annum than Māori. Possible reasons include less-severe illness for non-Māori, better focussed treatment for non-Māori, and greater supports and willingness to manage risk issues in the community for non-Māori. A qualitative study assessing the perspectives of Māori receiving CTO treatment would help understand these disparities further. In addition, a cultural lens on health care design and delivery is required to mitigate and address these issues over time.

Important outcomes associated with CTO usage are psychiatric admissions and community care contacts. In this analysis, we examined these outcomes according to age, deprivation and ethnicity splits because of the known associations between these potential confounders and health outcomes. The analyses of admissions largely confirmed our previous findings of reduced admissions on CTOs for patients with Psychotic Disorders. Higher rates of community care on CTOs compared to off CTOs were observed for all comparisons. Clear patterns of response distinguishing between Māori and non-Māori were by and large not present.

A notable finding was that non-Māori are more likely to receive more additional depot antipsychotic medication on CTOs compared with off CTOs than Māori. This was present for all the age, deprivation, ethnicity and diagnosis comparisons. Treatment with depot antipsychotic medication is the core compulsory intervention enabled by CTOs. Depot antipsychotic medication can be distinguished from treatment with oral medication which requires patient agreement for treatment to occur and is harder to monitor in the community. Treatment with depot antipsychotic medication is likely to be a key factor in the reduced rate of admissions we have previously reported on CTOs for patients with psychotic disorders (Beaglehole et al., 2021, 2022). Therefore, the juxtaposition of high rates of CTO usage for Māori and lower relative utilisation of depot antipsychotic treatment is concerning. It suggests that there may be systemic factors influencing Māori to be more likely to be exposed to the adverse impacts of compulsory treatment such as coercion and stigma but less likely to receive the benefits that may arise from treatment with antipsychotics.

Seven percent of the Māori cohort and 10% of the non-Māori cohort died during the 10-year study period. This high-mortality rate is probably a reflection of the underlying severity of the group receiving compulsory treatment. There were insufficient numbers to further analyse mortality data according to the age/deprivation/ethnicity groupings. Similarly, this prevented further analysis of mortality cause.

Our data include all New Zealanders placed on CTOs over a 10-year period and is therefore a comprehensive and inclusive data set. However, we endorse caution in the interpretation of real-world data. We have provided data allowing comparisons between Māori and non-Māori placed on CTOs to be made. We have attempted to critically analyse the findings using equity as the basis for discussion. Our positioning as a mixed New Zealand European (Pākeha) and Māori research authorship group provides the backdrop for our interpretations, and we encourage alternative opinions based on the data we present.

The issues raised in this report are not unique to New Zealand and have also been reported for indigenous populations in Australia (Kisely et al., 2020). Our findings suggest a disconnect between Māori service users, and the services that are currently available to them for assessing and managing mental disorders. The measurement and highlighting of disparities in this report is only the first step to addressing these concerns. Ongoing monitoring of CTO outcomes is required to track progress. The changing patterns of mental illness for Māori outlined in our introduction suggest inequities have emerged over time and are not immutable. Further research is desirable to investigate strategies to mitigate inequity. We recommend the inclusion of cultural experts to oversee health care design and delivery as a means of challenging and addressing the systematic issues documented in this report. Public health advisories also recommend the importance of tino rangatiratanga or Māori self-determination in the design, delivery, and monitoring of health services (Public Health Advisory Committee, 2023). One of the Te Tiriti O Waitangi principles is active protection; this means acting to the fullest extent practicable to achieve equitable health outcomes for Māori. In keeping with this approach, we suggest that Kaupapa Māori health services (in which Māori knowledge, skills, attitudes and values are integral) are needed to offer alternative treatment options for Māori.

In conclusion, this study compared outcomes of CTO utilisation for Māori with non-Māori to consider whether CTO usage is equitable between ethnic groups. We believe that the differences we identify between Māori and non-Māori reflect the presence of structural inequity of mental illness burden in New Zealand. Our findings reinforce the importance of cultural expertise throughout health care to minimise the extent to which disparity between ethnic groups is heightened by bias and racism. We also encourage ongoing research in areas relating to CTOs as these are applied to vulnerable patients, overrule human rights, and consequently require high levels of scrutiny.

## Supplemental Material

sj-docx-1-anp-10.1177_00048674241280918 – Supplemental material for Compulsory Community Treatment Orders and health outcomes for Ma-ori in New Zealand Supplemental material, sj-docx-1-anp-10.1177_00048674241280918 for Compulsory Community Treatment Orders and health outcomes for Ma-ori in New Zealand by Ben Beaglehole, Chris Frampton, Giles Newton-Howes, Arahia Kirikiri and Cameron Lacey in Australian & New Zealand Journal of Psychiatry
