# Supplementary material for: Compulsory Community Treatment Orders and health outcomes for Ma-ori in New Zealand
Source: Aust N Z J Psychiatry. 2024 Sep 26;58(12):1047–54. doi: 10.1177/00048674241280918 (PMC11585180; doi:10.1177/00048674241280918)
Supplement: sj-docx-1-anp-10.1177_00048674241280918 – Supplemental material for Compulsory Community Treatment Orders and health outcomes for Ma-ori in New Zealand [file sj-docx-1-anp-10.1177_00048674241280918.docx]

**Supplementary material**

**Supplementary figure 1**

Rate ratios of community contacts on CTOs: off CTOs according to ethnicity, deprivation and age for Psychotic Disorders (error bars show 95% confidence intervals).

**Supplementary figure 2**

Rate ratios of community contacts on CTOs: off CTOs according to ethnicity, deprivation and age for non-Psychotic Disorders (error bars show 95% confidence intervals).

**Supplementary figure 3**

Rate ratios of antidepressant dispensing on CTOs: off CTOs according to ethnicity, deprivation and age for Psychotic Disorders (error bars show 95% confidence intervals).

**Supplementary figure 4**

Rate ratio of antidepressant dispensing on CTOs: off CTOs according to ethnicity, deprivation and age for non-Psychotic Disorders (error bars show 95% confidence intervals).

**Supplementary figure 5**

Rate ratios of anxiolytic dispensing on CTOs: off CTOs according to ethnicity, deprivation and age for Psychotic Disorders (error bars show 95% confidence intervals).

**Supplementary figure 6**

Rate ratios of anxiolytic dispensing on CTOs: off CTOs according to ethnicity, deprivation and age for non-Psychotic Disorders (error bars show 95% confidence intervals).

**Supplementary figure 7**

Rate ratios of oral antipsychotic dispensing on CTOs: off CTOs according to ethnicity, deprivation and age for Psychotic Disorders (error bars show 95% confidence intervals).

**Supplementary figure 8**

Rate ratios of oral antipsychotic dispensing on CTOs: off CTOs according to ethnicity, deprivation and age for non-Psychotic Disorders (error bars show 95% confidence intervals).
